# *Bacillus amyloliquefaciens* MU2 fermentation enhances protein extraction and sensory quality of *Chlorella pyrenoidosa*

**DOI:** 10.1038/s41538-026-00949-7

**Published:** 2026-06-20

**Authors:** Kyung-Jin Cho, Min-Ung Kim, Do-Kyun Kim, Geum-Jae Jeong, Du-Min Jo, Seul-Ki Park, Minji Kim, Yoonsook Kim, Young-Mog Kim

**Affiliations:** 1 Functional Platform Research Group, Korea Food Research Institute, Wanju-gun, Republic of Korea; 2 Department of Food Science and Technology, Pukyong National University, Busan, Republic of Korea; 3 National Marine Biodiversity Institute of Korea, Seochun, Republic of Korea; 4 Smart Manufacturing Research Group, Korea Food Research Institute, Wanju-gun, Republic of Korea; 5 Research Center for Marine Integrated Bionics Technology, Pukyong National University, Busan, Republic of Korea

**Keywords:** Biochemistry, Biotechnology, Microbiology

## Abstract

The global demand for sustainable, nutritious protein sources necessitates innovative production strategies. *Chlorella pyrenoidosa* exhibits potential as a protein source; however, conventional extraction methods are limited in terms of protein extraction efficiency and product sensory attributes and quality. Microbial fermentation may address these challenges by improving protein extraction efficiency, antioxidant-related properties, sensory properties, and facilitating the production of improved microalgal protein materials. We developed a method for producing protein-rich materials through fermentation of *C. pyrenoidosa* with *Bacillus amyloliquefaciens* MU2 and optimized the fermentation conditions. Under the optimized conditions (26.6 h, 33.8 °C, and 2.7% inoculum), the predicted protein extraction efficiency was 42.8%, and the actual value was 42.90%. Fermentation enhanced the nutritional and functional qualities of *Chlorella* proteins and effectively mitigated undesirable flavors. In particular, crude protein content increased from 58.47 ± 0.27% to 78.10 ± 0.20%, while DPPH and ABTS radical scavenging activities increased from 5.01 ± 0.13% to 39.38 ± 0.37% and from 3.10 ± 0.10% to 47.89 ± 0.06%, respectively. Semi-pilot experiments demonstrated similar improvement trends, supporting the feasibility of the proposed process. These results suggest that fermentation with *B. amyloliquefaciens* MU2 is a promising strategy for improving the extraction efficiency and quality of *C. pyrenoidosa*-derived protein materials.

## Introduction

Global concerns regarding food security are increasing due to population growth, natural resource depletion, and anthropogenic environmental changes^1^. Consistent with worsening indicators of food insecurity and the growing burden of malnutrition worldwide^2–6^, the demand for sustainable protein sources continues to rise^7^. Modern agriculture and food production encounter challenges related to substantial resources, high energy consumption, and greenhouse gas emissions associated with animal protein production^8^. Consequently, the interest in alternative protein sources, such as plants and industrial byproducts, is increasing. Microalgal biomass, particularly from *Chlorella pyrenoidosa*, represents an attractive source for sustainable protein production. It is particularly rich in protein and exhibits a well-balanced amino acid profile, containing all essential and non-essential amino acids, as well as vitamins and minerals^9^. Additionally, *C. pyrenoidosa* can be cultivated with relatively low resource input and has been reported to show effective CO_2_ fixation capacity^10,11^. However, the rigid, multilayered cell wall of *C. pyrenoidosa* is highly resistant to mechanical and chemical disruption, making protein extraction difficult and ultimately limiting bioavailability^12^. Overcoming this barrier is crucial for improving the utilization of *C. pyrenoidosa* as a protein source^13^. Industrial-scale physical methods such as bead milling^14^ and high-pressure homogenization^15^ remain the main approaches for disrupting *Chlorella* spp., but they involve high energy consumption and are difficult to implement at large scale. Previous studies have shown that non-biological approaches, including alkaline treatment, bead milling, and salting-out/alkaline extraction, can improve protein extraction from *Chlorella* spp., with reported values such as 33.2% after alkaline treatment, 40% by bead milling alone, and 62% under salting-out/alkaline conditions^13,16,17^. Chemical extraction (organic solvents, strong alkali/acid) can increase yields, yet co-extracts pigments and lipids, complicating downstream purification^18^. More importantly for food applications, harsh lysis and solvent exposure can promote the formation or release of volatile aldehydes and alcohols, thereby increasing off-notes, such as green, grassy, or fishy odors, that impair sensory quality^19^. Thus, although these approaches are effective for improving extraction, they are primarily extraction-oriented. Accordingly, a mild processing strategy that can improve both protein extraction efficiency and sensory quality is needed for the food utilization of *C. pyrenoidosa*.

Microbial fermentation may enhance protein availability and the taste and flavor of microalgal proteins through enzymatic degradation of the cell wall and related structural components^20^. Additionally, as a sustainable and highly selective process that operates under mild reaction conditions, fermentation remains an attractive alternative to conventional chemical and physical protein extraction methods when both extractability and food-relevant quality improvement are considered^21^. The cell wall of *C. pyrenoidosa* is primarily composed of cellulose and hemicellulose, with structural proteins serving as glycan chain linkers^22^. Therefore, the combined action of cell wall-degrading and proteolytic enzymes may facilitate cell wall disruption and the release of intracellular proteins. *Bacillus* spp.-derived enzymes can decompose these structural proteins and glycan structures, improving the efficiency of protein extraction from *C. pyrenoidosa*^23^. In particular, *Bacillus amyloliquefaciens* is one of the most significant industrial microorganisms, known for producing degradative enzymes, such as cellulase, protease, and glucanase^24^. Several studies have reported that *B. amyloliquefaciens* improves the nutritional quality of protein-rich plant substrates, including soybean-based materials and rice beans, by promoting protein hydrolysis and increasing soluble protein or amino acid levels^25–28^. In addition, fermentation strategies have shown potential for improving the utilization and quality of microalgal biomass. These findings indicate that *B. amyloliquefaciens* is a suitable candidate for improving protein extraction efficiency and product quality during *C. pyrenoidosa* fermentation.

In our previous studies, enzymatic extraction demonstrated the potential to improve protein extraction efficiency from *C. pyrenoidosa*^23^. Additionally, enzyme-producing microorganisms were isolated from Jeotgal, and their enzymatic activities and safety were evaluated^29^. Among the tested isolates*, B. amyloliquefaciens* MU2 showed the highest protein extraction efficiency during *C. pyrenoidosa* fermentation and was therefore selected for the present study^29^. Based on its multiple enzyme activities, we hypothesized that fermentation with MU2 would promote cell wall degradation and intracellular protein release, thereby improving protein extraction efficiency as well as the physicochemical, nutritional, and sensory properties of the fermented product. To test this hypothesis, this study evaluated whether fermentation with *B. amyloliquefaciens* MU2 could promote cell wall disruption and protein release from *C. pyrenoidosa*, while improving its physicochemical, nutritional, and sensory properties. In addition, fermentation conditions were optimized, and the feasibility of semi-pilot-scale production was evaluated.

## Results

### Screening of the optimal culture medium for *C. pyrenoidosa* fermentation

**Fig. 1:**
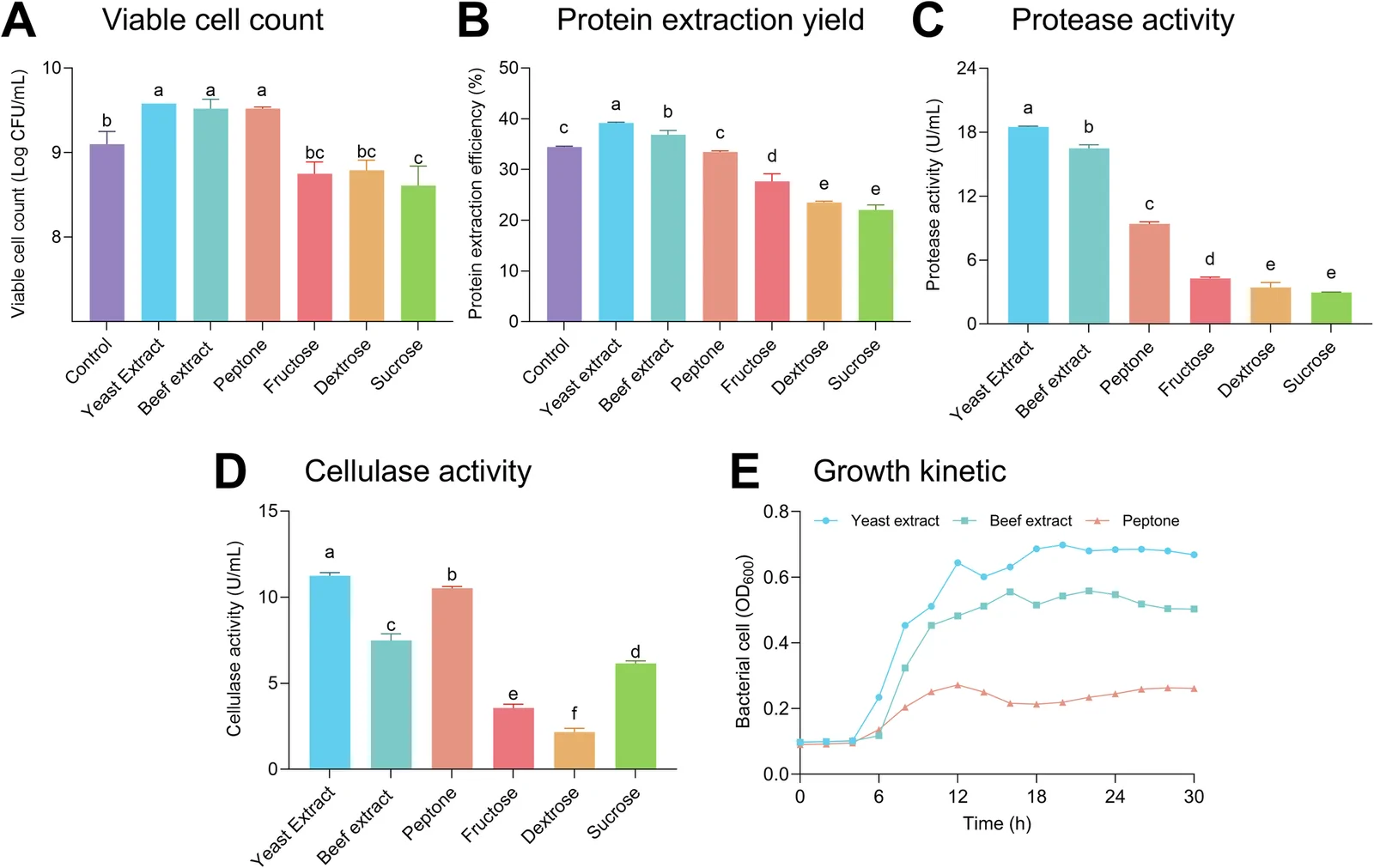
Effect of carbon and nitrogen sources (1% w/v) on protein extraction from *C.pyrenoidosa* fermented with *B. amyloliquefaciens* MU2. **A** Viable cell count (log CFU/mL), **B** protein extraction efficiency (%), **C** protease activity (U/mL), **D** cellulase activity (U/mL), and **E** growth kinetics. Different letters indicate significant differences (*p* < 0.05).

Figure 1 shows the effects of different nitrogen and carbon sources on *B. amyloliquefaciens* MU2 fermentation, including viable cell count, protein extraction efficiency from *C. pyrenoidosa*, protease activity, cellulase activity and growth kinetics. The viable cell count was higher in media supplemented with nitrogen sources than in those containing only carbon sources (Fig. 1A). In particular, in media containing yeast extract, beef extract, or peptone, the viable cell count reached 9.57 ± 0.15, 9.58 ± 0.02, and 9.52 ± 0.02 log CFU/mL, respectively. In contrast, in media with fructose, dextrose, or sucrose as carbon sources, it was 8.75 ± 0.14, 8.79 ± 0.12, and 8.61 ± 0.23 log CFU/mL, respectively, and these values were lower than those in the control group (9.10 ± 0.15 log CFU/mL). Protein extraction efficiency was the highest in yeast extract medium (39.20 ± 0.11%) (Fig. 1B). When beef extract or peptone was used as a nitrogen source, the protein extraction efficiencies were also notably high (36.84 ± 0.85 and 33.43 ± 0.25%, respectively). Conversely, media with fructose, dextrose, or sucrose produced lower efficiency than the control. Protease and cellulase activities were also higher in nitrogen-source media, showing trends generally similar to that of protein extraction efficiency (Fig. 1C, D). Protease activity was highest in yeast extract medium (18.50 ± 0.08 U/mL), followed by beef extract (16.48 ± 0.35 U/mL) and peptone (9.39 ± 0.20 U/mL), whereas fructose, dextrose, and sucrose showed lower activities of 4.29 ± 0.12, 3.43 ± 0.47, and 2.96 ± 0.04 U/mL, respectively. Likewise, cellulase activity was highest in yeast extract medium (11.25 ± 0.18 U/mL), followed by peptone (10.52 ± 0.10 U/mL) and beef extract (7.48 ± 0.39 U/mL), whereas sucrose, fructose, and dextrose showed lower activities of 6.14 ± 0.16, 3.56 ± 0.22, and 2.18 ± 0.21 U/mL, respectively. Although the rank order of the nitrogen sources was not completely identical between the two enzymes, nitrogen-source conditions consistently promoted stronger extracellular enzyme activities than carbon-source conditions under the tested screening conditions. *B. amyloliquefaciens* MU2 cultured in yeast extract reached the exponential and stationary phases more rapidly than when cultured with other nitrogen sources (Fig. 1E). Based on these results, yeast extract was selected as the basal nitrogen source for subsequent optimization of fermentation conditions.

### Optimization of *B. amyloliquefaciens* MU2 fermentation conditions

**Fig. 2:**
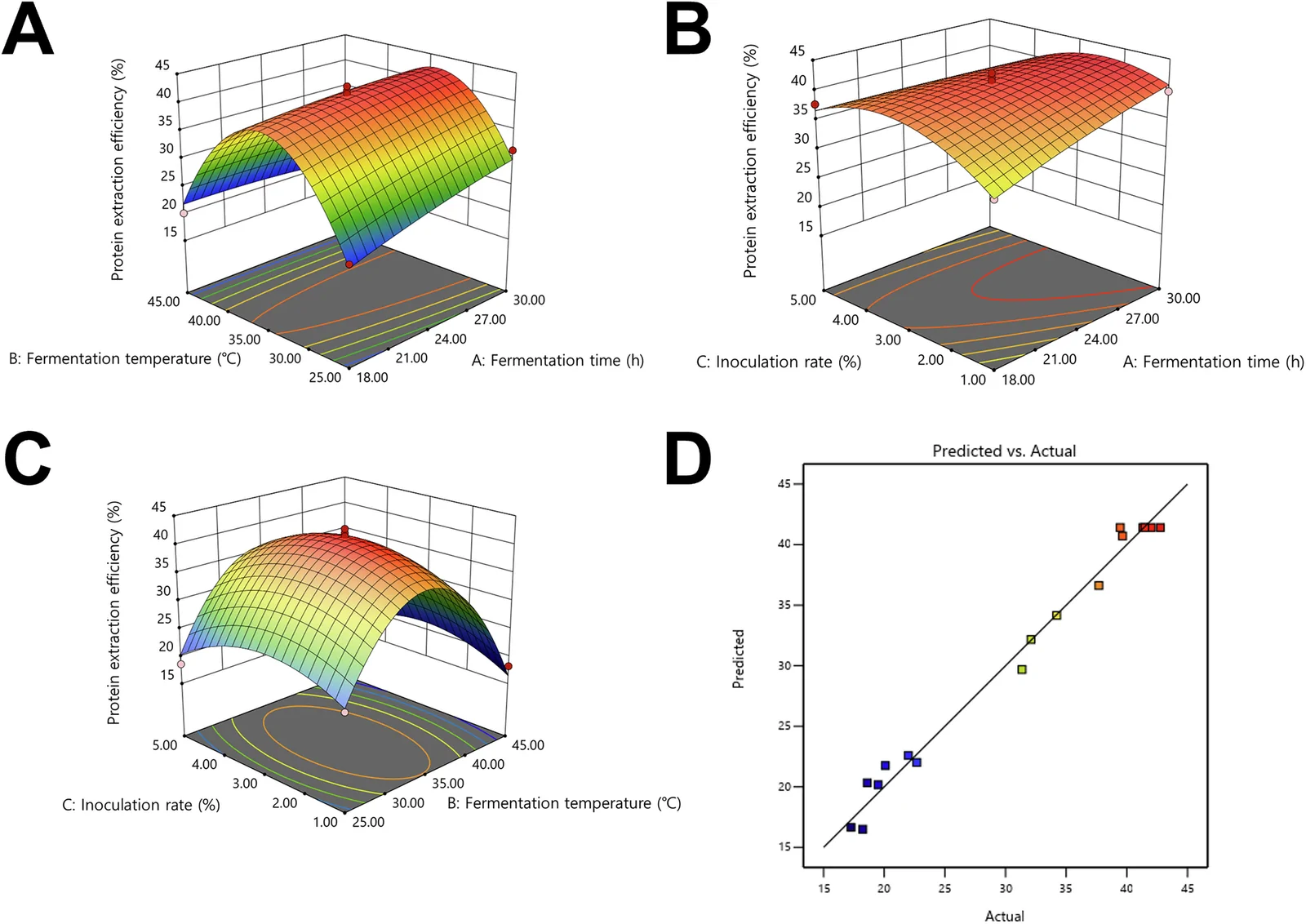
Three-dimensional response surface plots for protein extraction efficiency. The plots showing the effects of **A** fermentation time (h) and fermentation temperature (°C), **B** fermentation time (h) and inoculation rate (%), **C** fermentation temperature (°C) and inoculation rate (%) on protein extraction efficiency (%) from *C. pyrenoidosa*, and **D** the predicted versus actual values.

The optimal fermentation conditions were predicted using a second-order polynomial equation based on variance analysis, resulting in 17 predicted values for *B. amyloliquefaciens* MU2 fermentation conditions (Table S1). Analysis of variance results for the second-order model are presented in Table S2, and the regression analysis is summarized in Table S3. The quadratic model was significant (*F* = 24.78, *p* < 0.0001), whereas the lack of fit was not significant (*p* = 0.1327)^30,31^. The model also showed a high coefficient of determination (*R*^2^ = 0.9864; adjusted *R*^2^ = 0.9689) and an adequate precision value of 18.53^32^. The independent variables *A* (h), *B* (°C), and *C* (%) were statistically significant (*p* < 0.05). Among the second-order terms, *B*^2^ and *C*^2^ were statistically significant (*p* < 0.05), whereas *A*^2^ was not. Additionally, the interaction terms AB and AC were significant, whereas BC was not. These results were consistent with the response surface plots (Fig. 2A–C), in which the surfaces for AB and AC showed more pronounced curvature and interaction patterns than that for BC. The coded and uncoded forms of the fitted quadratic model are shown below.

Coded: Protein extraction efficiency (%) = 41.41 + 1.52 A − 2.44 B − 0.52 C − 2.32 AB − 2.75 AC + 0.61 BC − 0.55 A^2^ − 17.45 B^2^ − 4.94 C^2^

Uncoded: Protein extraction efficiency (%) = −234.76924 + 3.02826 A + 12.80408 B + 11.59532 C − 0.038587 AB − 0.22954 AC + 0.030369 BC − 0.015328 A^2^ − 0.17448 B^2^ − 1.23521 C^2^

The predicted-versus-actual plot is presented in Fig. 2D. Model adequacy was further assessed using residual diagnostic plots (Fig. S2). The residuals were approximately normally distributed and did not show obvious systematic patterns with predicted values or run order, supporting the adequacy of the fitted model. The optimal fermentation conditions for *B. amyloliquefaciens* MU2 to achieve the highest *C. pyrenoidosa* protein extraction efficiency based on the RSM-BBD results are detailed in Table S4. The optimal conditions were as follows: *A* = 26.6 h, *B* = 33.8°C, and *C* = 2.7%. The predicted (42.98%) protein extraction efficiency closely matched the actual value (42.90%), with no statistically significant difference (*p* > 0.05). The optimized fermentation products were used for subsequent compositional, functional, and sensory comparisons between the unfermented *C. pyrenoidosa* biomass (unFCP) and the supernatant fraction of fermented *C. pyrenoidosa* (FCP).

### Structural and compositional properties of unFCP and FCP

**Fig. 3:**
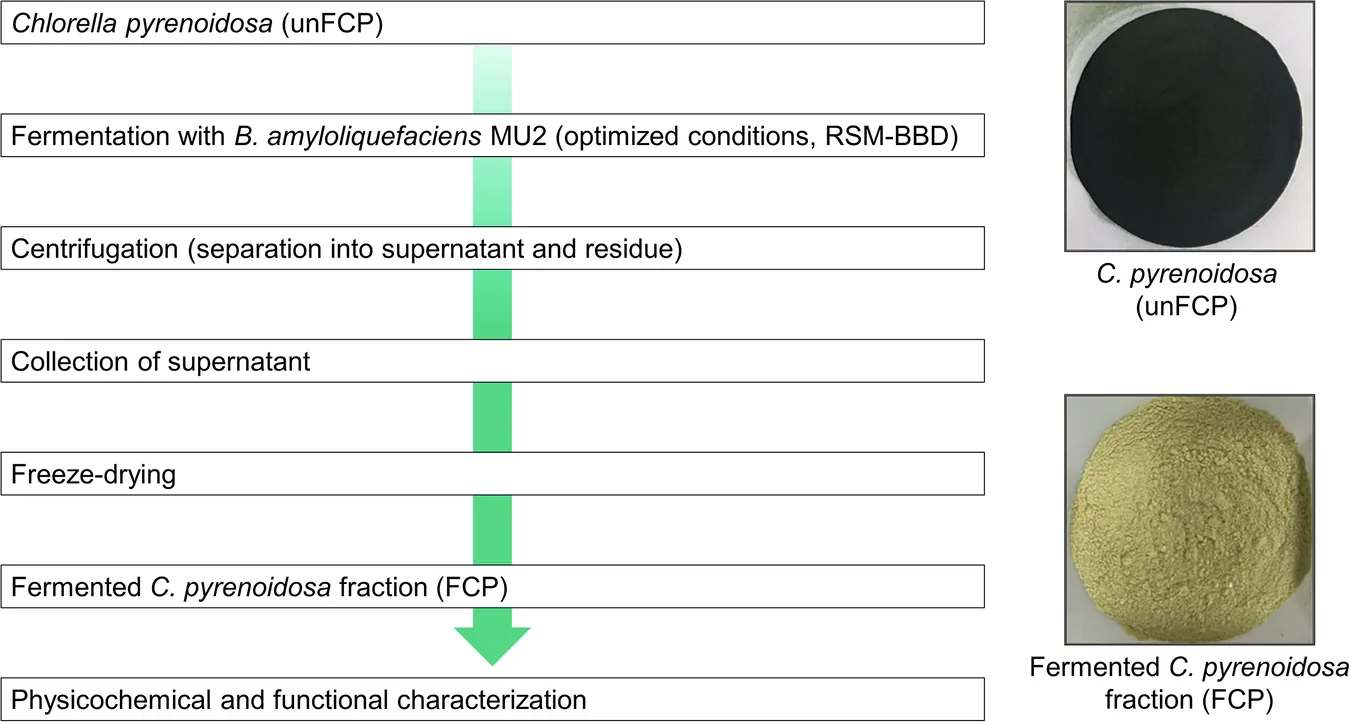
Experimental workflow of the study. Schematic overview of the preparation of FCP from unFCP. The arrow indicates the processing sequence, and the images show unFCP and FCP.

To determine how fermentation affected the structural and compositional characteristics of the analyzed fractions, we compared the microstructure, hydrolysis behavior, protein profiles, and compositions of unFCP and FCP according to the workflow shown in Fig. 3.

**Fig. 4:**
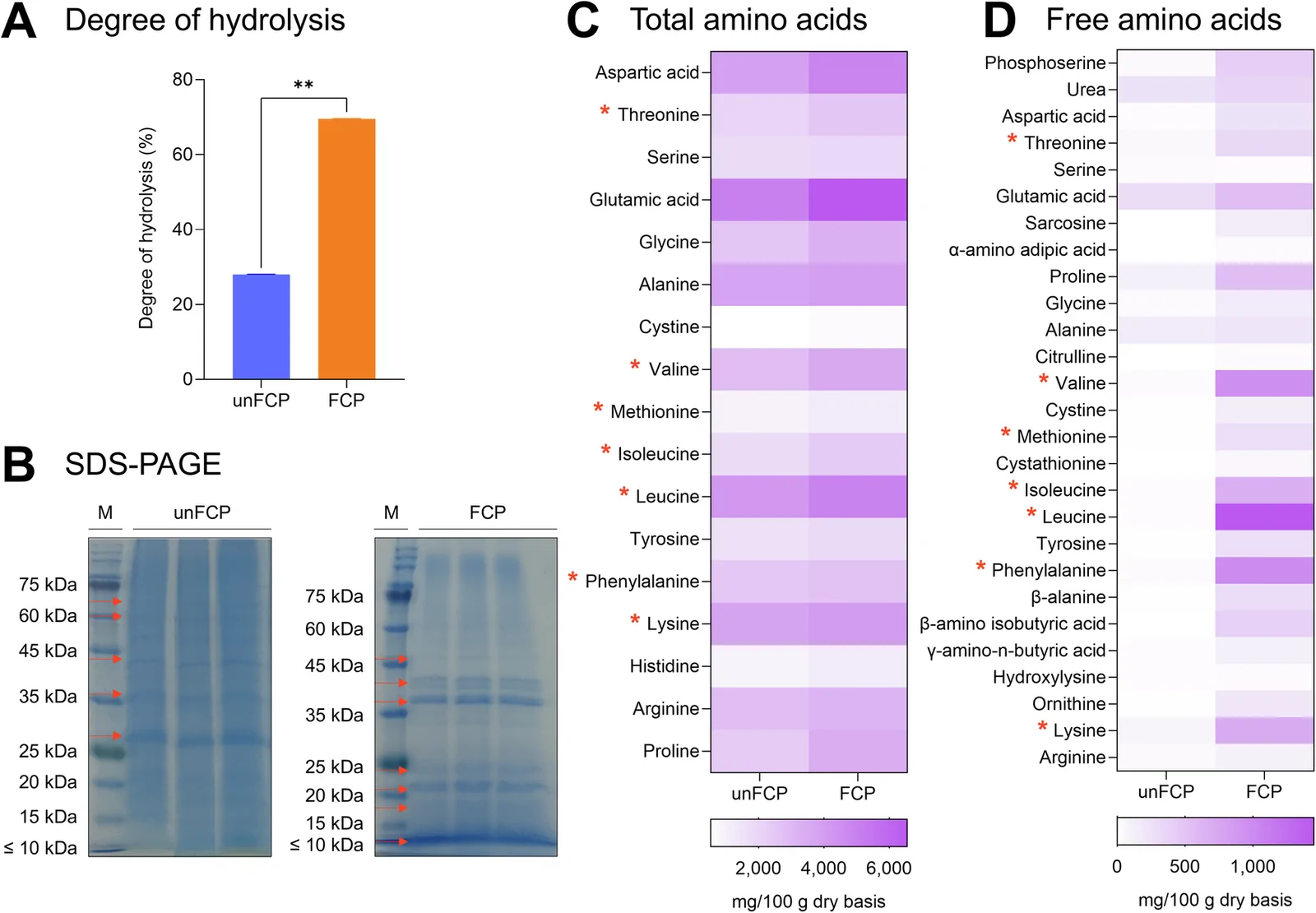
Protein characterization and amino acid profiling heatmap of unFCP and FCP. **A** DH, **B** SDS-PAGE analysis of protein fractions, **C** total amino acid content, and **D** free amino acid content. M denotes the protein molecular mass marker (10–245 kDa). A red asterisk denotes essential amino acids. Statistical significance between unFCP and FCP was assessed using an independent samples t-test, where **p < 0.01.

The DH values of unFCP and FCP were 28.00 ± 0.05% and 69.56 ± 0.06%, respectively (Fig. 4A). To examine the molecular weight distribution, the unFCP and FCP protein samples were analyzed by sodium dodecyl sulfate-polyacrylamide gel electrophoresis (SDS-PAGE) (Fig. 4B). For unFCP, bands were observed around 63, 60, 43, 36, and 27 kDa, whereas FCP produced bands around 49, 42, 37, 24, 21, 19, and <10 kDa, indicating the breakdown of *C. pyrenoidosa* proteins into smaller fragments through microbial fermentation.

The proximate components of unFCP and FCP are shown in Table S5. Compared to unFCP, FCP had higher crude protein, ash, and moisture contents, whereas carbohydrate and crude fat levels were lower.

The total and free amino acid contents of unFCP and FCP are provided in Tables S6 and S7. The contents of 17 amino acids, except serine, increased after fermentation (Fig. 4C). In unFCP, the essential amino acid (EAA) and total amino acid (TAA) contents were 17,917.9 ± 118.9 and 43,034.8 ± 69.8 mg/100 g, respectively, whereas in FCP, these values significantly increased to 21,232.5 ± 252.4 and 51,846.5 ± 610.3 mg/100 g, respectively. The free essential amino acid and total free amino acid contents also increased markedly after fermentation, from 210.1 ± 0.3 and 1192.5 ± 8.7 mg/100 g in unFCP to 5363.8 ± 151.7 and 9617.9 ± 215.2 mg/100 g in FCP, respectively.

**Fig. 5:**
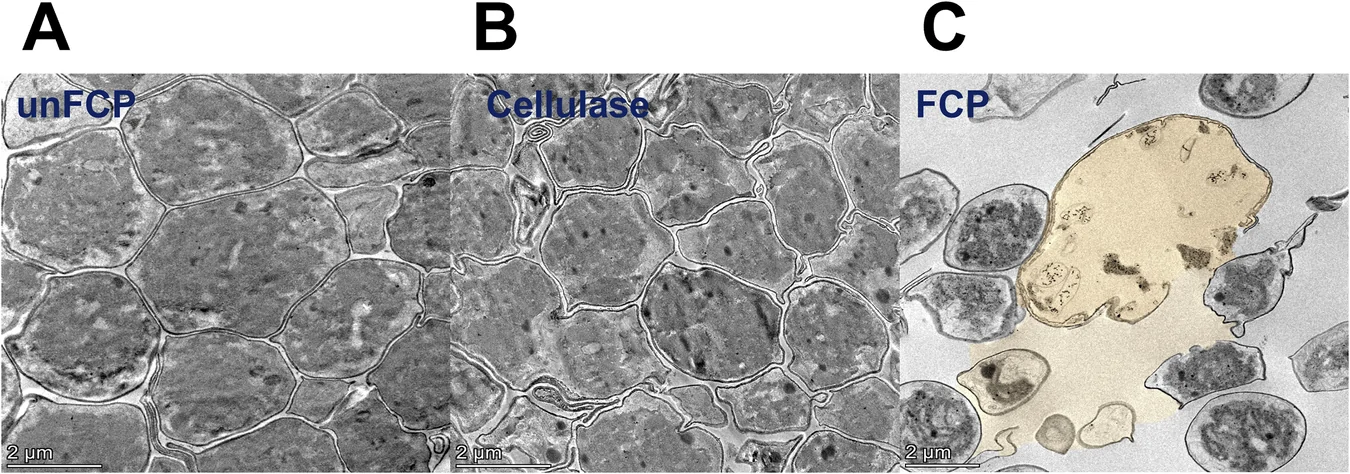
Bio-TEM images of *C.pyrenoidosa* under different treatment conditions. **A** unFCP showing intact cell walls and dense intracellular structures, **B** cellulase-treated sample showing no distinct structural difference compared with unFCP, **C** FCP showing disrupted cell walls and cellular debris. Scale bars, 2 μm.

Bio-TEM observation further supported the structural effect of fermentation on *C. pyrenoidosa* (Fig. 5). unFCP showed intact cell walls and dense intracellular structures (Fig. 5A). The cellulase-treated sample did not show a distinct structural difference compared with unFCP (Fig. 5B). In contrast, FCP showed disrupted cell walls and cellular debris, indicating that fermentation with *B. amyloliquefaciens* MU2 was associated with marked structural alteration of the biomass (Fig. 5C).

### Antioxidant activities and total phenolic content (TPC) of unFCP and FCP

**Fig. 6:**
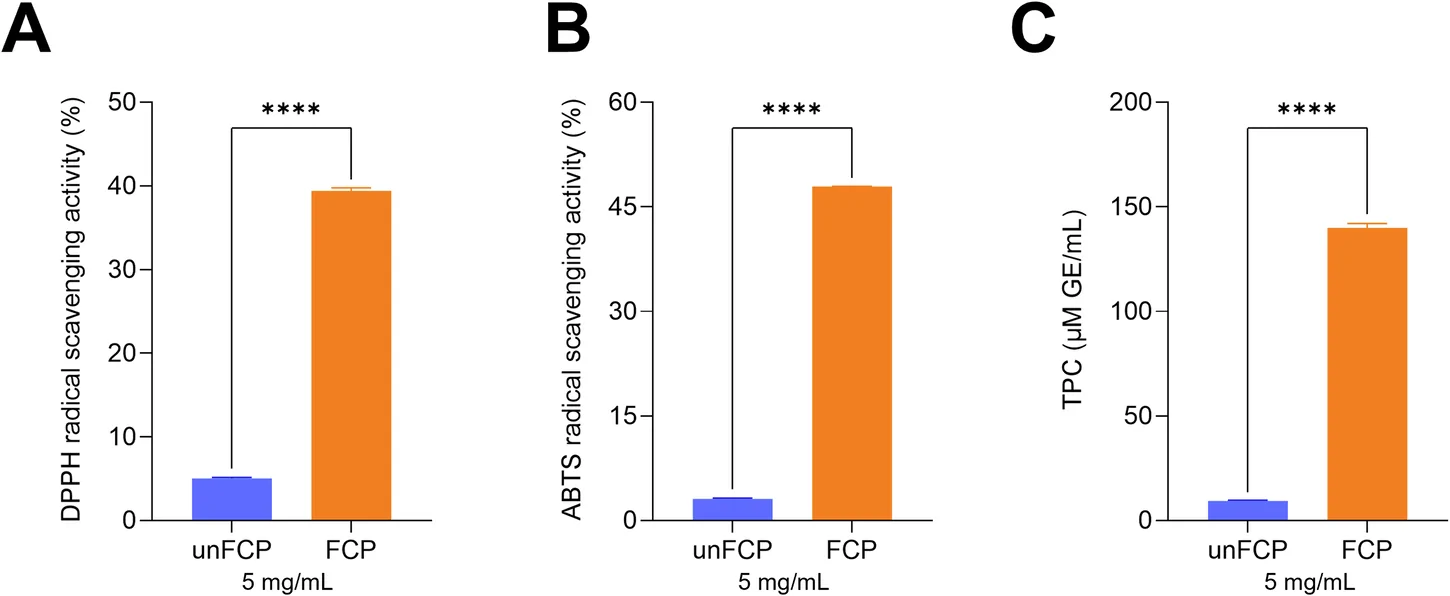
Antioxidant capacity and TPC of unFCP and FCP. **A** DPPH assay results, **B** ABTS assay results, and **C** TPC. Values are mean ± SD (*N* = 3). Statistical significance between unFCP and FCP was assessed using an independent samples t-test, where ****p < 0.0001.

The 2,2-diphenyl-1-picrylhydrazyl (DPPH) and 2,2-azino-bis(3-ethylbenz-thiazoline-6-sulfonic)acid (ABTS) radical scavenging activities, along with the TPC results of unFCP and FCP, are shown in Fig. 6. The DPPH radical scavenging activity was 5.01% ± 0.13% for unFCP and 39.38% ± 0.37% for FCP (*p* < 0.0001; Fig. 6A). The ABTS radical scavenging activity was 3.10% ± 0.1% for unFCP and 47.89% ± 0.06% for FCP (*p* < 0.0001; Fig. 6B). The TPCs for unFCP and FCP were 9.34 ± 0.37 and 139.95 ± 2.11 µM GAE/mL, respectively (*p* < 0.0001; Fig. 6C). Notably, the DPPH, ABTS, and TPC values of FCP were approximately 8, 15, and 15 times higher, respectively, than those of unFCP.

### Sensory properties of unFCP and FCP

**Fig. 7:**
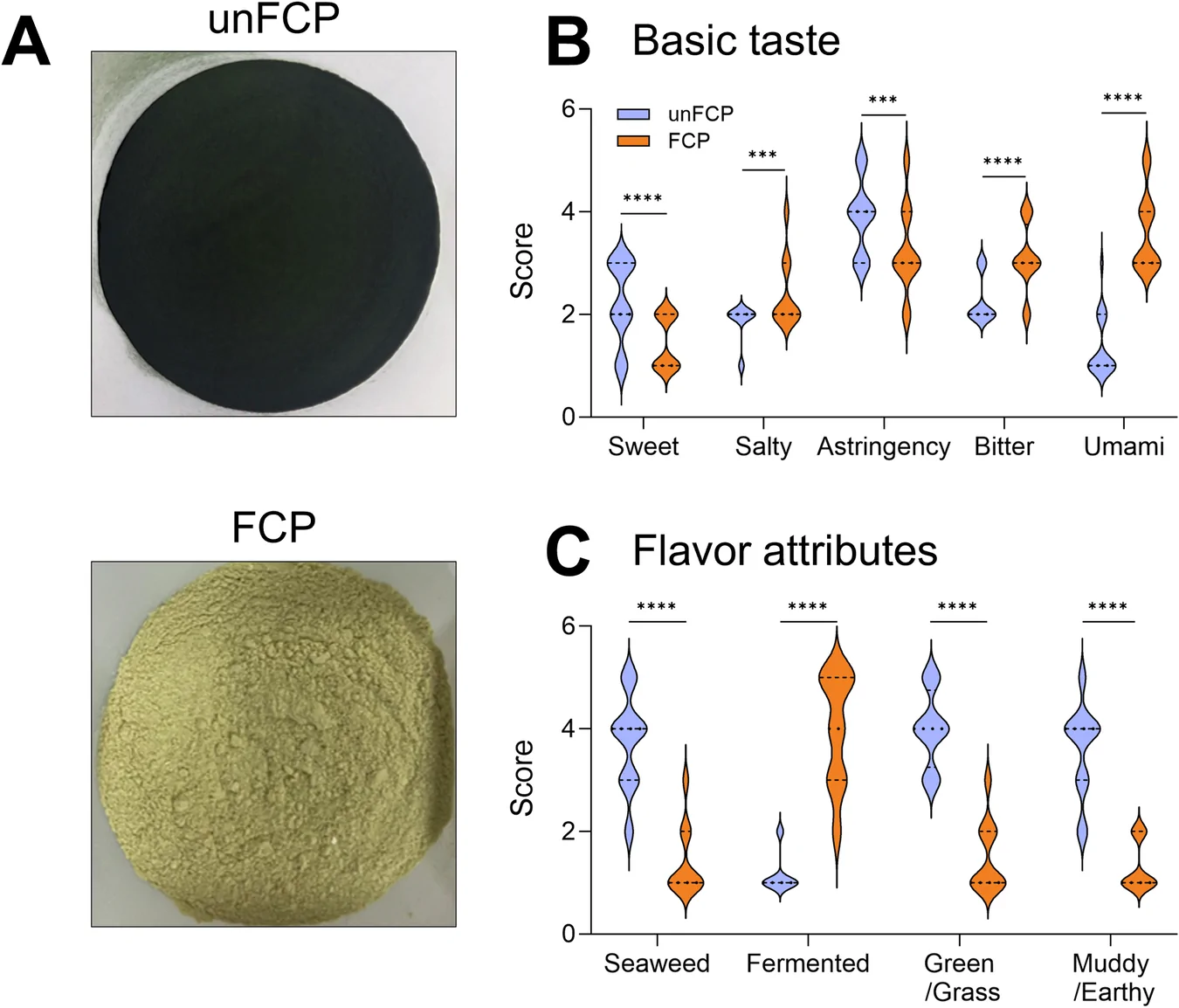
Representative images and violin plots of sensory evaluation of unFCP and FCP. **A** Images of unFCP and FCP, **B** basic tastes, **C** flavor attributes sensory evaluation chart (*n* = 20). Sensory scores were recorded on a 1–5 intensity scale; the violin shape represents a smoothed distribution and does not indicate observed values outside the actual scoring range. Statistical significance between unFCP and FCP was assessed using an independent samples t-test, where ***p < 0.001, and ****p < 0.0001.

Table 1 shows the volatile organic compounds (VOCs) present in unFCP and FCP. unFCP contained 15 compounds, including 3 alcohols, 6 aldehydes, 2 ketones, 1 ester, 2 furans, and 2 siloxanes. FCP contained 32 compounds, including 10 alcohols, 3 aldehydes, 10 ketones, 3 esters, 3 acids, 2 sulfur compounds, and 1 other compound, indicating a substantial expansion of the VOC profile resulting from the fermentation process. Representative images of unFCP and FCP and the results of sensory evaluation for basic tastes and aroma attributes are shown in Fig. 7. After fermentation, the green color shifted to yellowish-white (Fig. 7A). FCP displayed distinct sensory characteristics from unFCP. The sensory score for sweetness decreased from 2.2 ± 0.8 to 1.4 ± 0.7, whereas the scores for bitterness and umami increased from 2.2 ± 0.6 to 3.2 ± 0.8 and from 1.2 ± 0.4 to 3.2 ± 0.8, respectively (Fig. 7B). Among the odor attributes, the intensities of green/grass and muddy/earthy notes were reduced after fermentation, whereas the intensity of fermented odor increased (Fig. 7C). These results indicate that fermentation modulated both the color and flavor profile of the *C. pyrenoidosa*.

**Table 1:** VOCs detected in supernatant of unFCP and FCP

Volatile organic compounds	unFCP	FCP	Molecular formula	Aroma quality
Alcohols	3*	10		
Isopropyl alcohol	**+**	**−**	C₃H₈O	alcoholic
Isobutanol	**+**	**−**	C_4_H_10_O	sweet, alcoholic
1-Propanol	**−**	**+**	C₃H₈O	sweet, fruity
Isobutyl alcohol	**−**	**+**	C₄H₁₀O	fruity, sour
1-Butanol	**−**	**+**	C₄H₁₀O	sweet, alcoholic
1-Pentanol	**−**	**+**	C_5_H₁_2_O	sweet
Ethyl vinyl carbinol	**+**	**-**	C₄H₈O	alcoholic
1-Penen-3-ol	**−**	**+**	C₅H₁₀O	fresh, grassy
Isoamyl alcohol	**−**	**+**	C₅H₁₂O	fruity
3-Hydroxy-3-methyl-2-butanone	**−**	**+**	C₅H₁₀O₂	buttery, sweet
Isobutenylcarbinol	**−**	**+**	C₅H₁₀O	alcoholic
1-Hexanol	**−**	**+**	C₆H₁₄O	green, grassy
2,3-Dimethylbutan-1-ol	**−**	**+**	C₆H₁₄O	mild, sweet
Aldehydes	7	3		
Acetaldehyde	**+**	**−**	C₂H₄O	pungent, fruity
Isobutanal	**+**	**−**	C₄H₈O	fruity, almond
Isobutyraldehyde	**−**	**+**	C₄H₈O	sharp, acrid
Butanal	**+**	**-**	C₄H₈O	pungent, aldehydic
Isovaleraldehyde	**+**	**+**	C₅H₁₀O	sour
2-Methyl butanal	**−**	**+**	C₅H₁₀O	fruity, nutty
2-Methyl butyraldehyde	**+**	**-**	C₅H₁₀O	fruity, almond
Pentanal	**+**	**-**	C₅H₁₀O	pungent, aldehydic
n-Hexanal	**+**	**-**	C₆H₁₂O	green, grass
Ketones	2	10		
2,3-Butanedione	**−**	**+**	C₄H₆O₂	buttery, creamy
2-Butanone	**+**	**-**	C₄H₈O	acetone
Methyl ethyl ketone	**−**	**+**	C₄H₈O	sweet
Methyl isopropyl ketone	**+**	**-**	C₅H₁₀O	strong acetone
Methyl ethyl ketone	**−**	**+**	C₄H₈O	sweet
3-Methyl-2-butanone	**−**	**+**	C₅H₁₀O	sweet
2-Pentanone	**−**	**+**	C₅H₁₀O	sweet
2,3-Dihydro-5-methyl-1,4-dioxin	**−**	**+**	C₅H₈O₂	mildly pungent
Acetoin	**−**	**+**	C₄H₈O₂	buttery, creamy
2-Hexanone	**−**	**+**	C₆H₁₂O	green, grassy
2-Heptanone	**−**	**+**	C₇H₁₄O	sweet, fruity
Esters	1	3		
Methyl glycolate	−	+	C₃H₆O₃	sweet, fresh
Methyl acetate	+	+	C₄H₈O₂	sweet, fruity
Ethyl lactate	−	+	C₅H₁₀O₃	sweet, creamy
Furans	2	0		
2-Methyl furan	**+**	**−**	C₅H₆O	sweet, nutty
2-Ethylfuran	+	−	C₆H₈O	smoky burnt odor
Acids	0	**3**		
Acetic acid	−	+	C₂H₄O₂	sour, pungent
Isobutyric acid	−	+	C₄H₁₀O	fruity, sour
Butanoic acid	−	+	C₄H₈O₂	Fermented
Sulfur compounds	0	2		
Sulfur dioxide	−	+	SO₂	Pungent
Ethyl n-butyl disulfide	−	+	C₆H₁₄S₂	garlic, onion like
Aromatic hydrocarbons	1	0		
Toluene	+	−	C₇H₈	paint thinner like
Siloxanes	2	1		
Hexamethylcyclotrisiloxane	+	+	C₆H₁₈O₃Si₃	almost no smell
Octamethyl cyclo tetrasiloxane	+	+	C₈H₂₄O₄Si₄	almost no smell
Alkanes	1	0		
2,2,6-Trimethyldecane	+	−	C₁₃H₂₈	mild, chemical
Others	0	1		
2-Methylpyrazine	−	+	C₅H₆N₂	nutty, roasted

*Number of VOCs detected, + detected, − non-detected.

### Semi-pilot experiment of fermentation for protein extraction

Table 2 presents the results of *C. pyrenoidosa* protein extraction at different fermentation scales. The crude protein content was 78.10% ± 0.20% at the lab scale and 78.50% ± 0.10% at the semi-pilot scale. The extraction efficiencies were 42.90% ± 0.10% and 43.20% ± 0.05%, with recoveries of 57.50% ± 0.87% and 59.90% ± 1.35%, respectively. Although total EAA and total amino acid contents did not differ significantly between the lab-scale and semi-pilot-scale samples, the semi-pilot-scale sample showed significantly higher free EAA and total free amino acid contents, which increased from 5,363.8 ± 151.7 to 6,140.7 ± 22.1 mg/100 g (*p* = 0.017) and from 9,617.9 ± 215.2 to 10,738.6 ± 125.1 mg/100 g (*p* = 0.015), respectively (Tables 3 and 4). No significant difference was observed between the lab-scale and semi-pilot-scale fermentations in crude protein content or weight recovery rate (*p* > 0.05), whereas protein extraction efficiency was slightly but significantly higher at the semi-pilot scale (*p* = 0.009). Overall, the semi-pilot scale process showed broadly comparable performance to the lab-scale process, although a modest increase in protein extraction efficiency was observed under the semi-pilot condition.

**Table 2:** Protein extraction efficiency of *C. pyrenoidosa* according to fermentation capacity

Category	Lab scale	Semi-pilot scale	*t-*value	*p-*value
Fermentation volume	10 g / 200 mL	150 g / 3 L		
Crude protein (g/100 g dry basis)	78.10 ± 0.20	78.50 ± 0.10	−3.464	0.074
Protein extraction efficiency (%)	42.90 ± 0.10	43.20 ± 0.05	−10.392	0.009
Weight recovery rate^a^ (%)	57.50 ± 0.87	59.90 ± 1.35	−1.872	0.202

^a^Weight of recovered supernatant fermented *C. pyrenoidosa* (g) / Weight of initial *C. pyrenoidosa* (g).

**Table 3:** Total amino acid contents of *C. pyrenoidosa* according to fermentation capacity

Amino acids	Concentration of total amino acid (mg/100 g dry basis)		*t-*value	*p-*value
	Lab scale	Semi-pilot scale		
Aspartic acid	4892.9 ± 32.9	4611.7 ± 74.9	5.090	0.036
Threonine^*^	2552.2 ± 35.1	2635.9 ± 19.2	−4.762	0.041
Serine	1868.1 ± 54.2	2082.2 ± 77.1	−5.409	0.033
Glutamic acid	6539.8 ± 35.6	6966.8 ± 33.6	−21.349	0.002
Glycine	3234.9 ± 61.4	3259.7 ± 50.3	−0.443	0.701
Alanine	3903.4 ± 41.2	4052.6 ± 16.1	−5.043	0.037
Cystine	625.0 ± 14.5	517.8 ± 7.3	9.684	0.010
Valine^*^	3538.3 ± 26.5	3425.5 ± 3.8	7.869	0.016
Methionine^*^	1208.5 ± 18.3	1272.9 ± 50.9	−1.796	0.214
Isoleucine^*^	2320.9 ± 33.3	2358.3 ± 38.3	−1.044	0.406
Leucine^*^	4919.0 ± 42.1	4391.3 ± 23.5	25.012	0.002
Tyrosine	1798.7 ± 21.2	1976.4 ± 161.4	−2.026	0.180
Phenylalanine^*^	2677.1 ± 47.1	2859.3 ± 245.6	−1.398	0.297
Lysine^*^	4016.5 ± 50.0	3906.2 ± 130.3	1.679	0.235
Histidine	1190.4 ± 12.1	1332.5 ± 50.9	−4.250	0.051
Arginine	3159.7 ± 41.3	3533.2 ± 293.6	−2.466	0.133
Proline	3401.1 ± 43.5	2903.5 ± 114.6	6.095	0.026
∑EAA	21232.5 ± 252.4	20849.3 ± 333.0	2.206	0.158
∑NEAA	30614.0 ± 357.9	31236.2 ± 255.1	−5.213	0.035
TAA	51846.5 ± 610.3	52085.5 ± 574.0	−0.841	0.489

*Essential amino acids.

**Table 4:** Free amino acid contents of *C. pyrenoidosa* according to fermentation capacity

Amino acids	Concentration of total amino acid (mg/100 g dry basis)		*t-*value	*p-*value
	Lab scale	Semi-pilot scale		
Phosphoserine	418.8 ± 3.5	320.8 ± 8.5	23.037	0.002
Urea	357.6 ± 3.8	446.2 ± 45.8	−3.491	0.073
Aspartic acid	232.0 ± 1.8	335.4 ± 5.1	−40.672	0.001
Threonine^*^	332.3 ± 1.3	393.4 ± 7.5	−15.245	0.004
Serine	11.3 ± 0.0	11.1 ± 0.2	2.646	0.118
Glutamic acid	548.1 ± 10.7	644.2 ± 25.2	−7.598	0.017
Sarcosine	151.4 ± 9.6	227.7 ± 5.8	−23.269	0.002
α-amino adipic acid	20.3 ± 0.6	264.5 ± 19.8	−4.970	0.038
Proline	555.5 ± 5.4	512.2 ± 1.6	−45.634	0.000
Glycine	154.6 ± 4.9	227.7 ± 5.28	−8.640	0.013
Alanine	207.5 ± 20.0	264.5 ± 19.8	−101.757	0.000
Citrulline	28.9 ± 1.0	87.8 ± 2.6	−6.891	0.020
Valine^*^	950.6 ± 25.9	1074.9 ± 2.1	−11.050	0.008
Cystine	130.6 ± 0.8	196.6 ± 0.4	−30.178	0.001
Methionine^*^	270.8 ± 10.1	311.8 ± 0.4	−2.753	0.111
Cystathionine	54.6 ± 3.7	77.5 ± 0.3	−1.016	0.417
Isoleucine^*^	676.5 ± 14.7	909.8 ± 3.2	2.691	0.115
Leucine^*^	1447.8 ± 107.0	1617.0 ± 1.1	5.193	0.035
Tyrosine	267.5 ± 20.0	279.2 ± 0.1	24.785	0.002
Phenylalanine^*^	980.8 ± 16.6	954.7 ± 0.4	2.183	0.161
β-alanine	287.2 ± 15.4	239.6 ± 0.9	6.457	0.023
β-Amino isobutyric acid	391.7 ± 7.6	279.2 ± 0.5	−16.760	0.004
γ-Amino-n-butyric acid	115.7 ± 16.0	94.1 ± 5.9	−25.344	0.002
Hydroxylysine	26.7 ± 2.7	21.2 ± 1.3	−28.096	0.001
Ornithine	191.3 ± 11.8	290.2 ± 5.7	11.882	0.007
Lysine^*^	704.9 ± 13.5	879.3 ± 9.0	23.037	0.002
Arginine	102.6 ± 3.5	270.6 ± 8.2	−3.491	0.073
∑EAA	5363.8 ± 151.7	6140.7 ± 22.1	−7.517	0.017
∑NEAA	4254.1 ± 65.5	4597.9 ± 103.0	−7.662	0.017
TAA	9617.9 ± 215.2	10738.6 ± 125.1	−8.091	0.015

*Essential amino acids.

## Discussion

The higher viable cell count in nitrogen-supplemented media is attributable to the abundant nitrogen supply, supporting cell growth. In particular, yeast extract promoted rapid proliferation and high protease and cellulase activities, which together likely facilitated breakdown of the *C. pyrenoidosa* cell wall and subsequent protein release. Nitrogen sources are essential for promoting cell growth, serving as the primary substrates for protein synthesis and the formation of cellular components. Moreover, they likely enhance enzyme activity by improving tricarboxylic acid cycle efficiency, thus accelerating cell growth and energy production^33^. Microorganisms produce enzymes, such as protease and cellulase, during the late exponential or stationary growth phase^34^. Similar tendencies have been reported in other Bacillus systems, in which organic nitrogen sources such as peptone or yeast extract positively influenced extracellular cellulase or protease production^34–36^. Glycoproteins in the cell wall of green microalgae help maintain structure, support environmental adaptation, and facilitate physiological functions^37^. Moreover, they strengthen the cell wall and help defend against external stress by interacting with polysaccharides. In conclusion, culturing *B. amyloliquefaciens* MU2 in yeast extract medium enhances protease and cellulase production, which likely facilitates degradation of the *C. pyrenoidosa* cell wall during fermentation, thereby improving protein extraction efficiency. Nevertheless, because a non-inoculated yeast extract control was not included in the present screening design, the relative contributions of enhanced microbial performance and possible medium-related effects on apparent protein solubility could not be fully distinguished.

To maximize extraction efficiency under controlled conditions, we next optimized fermentation time, temperature, and inoculum concentration using RSM-BBD. The significance of the quadratic model and the close agreement between predicted and experimental values indicate that RSM was effective for optimizing the fermentation conditions of *B. amyloliquefaciens* MU2. These factors support the robustness of the RSM approach for optimizing fermentation conditions. Importantly, the close agreement between predicted and actual protein extraction efficiencies indicates that the process window is stable enough to support controlled production of FCP. In addition, the residual diagnostic plots supported the adequacy of the fitted model, indicating that no major deviation from model assumptions was evident under the tested conditions (Fig. S2). The optimized MU2 fermentation process achieved a protein extraction efficiency of 42.9%, which was higher than some previously reported values for non-biological extraction of *Chlorella*, such as 33.2% after alkaline treatment, but lower than those reported for certain mechanically intensive or combined strategies, including 40% protein recovery by bead milling alone, 47.3% protein extraction yield by dry bead milling integrated with alkaline solubilization, and 62% extraction efficiency by a salting-out/alkaline approach for *C. pyrenoidosa*^13,16,17^. However, direct comparison should be interpreted with caution because extraction conditions, biomass states, and analytical endpoints differed among studies. Within this context, the present result suggests that fermentation alone can provide competitive extraction-related performance under biologically mild conditions. More importantly, the significance of the present process lies not only in improving extraction efficiency, but also in supporting food-relevant quality improvement without mechanically intensive disruption or alkaline-assisted extraction.

Despite its excellent nutritional value, *C. pyrenoidosa* is difficult for humans to digest^38^. Hydrolysis through microbial fermentation has been reported to enhance the digestibility of *Chlorella* protein^39^. The increase in DH after fermentation is attributed to the microbial proteases, which decompose peptide bonds, generating low-molecular-weight peptide fractions and free amino acids. Notably, the presence of protein bands < 10 kDa suggests that the proteins were degraded into low-molecular-weight peptides through the proteolytic action of *B. amyloliquefaciens* MU2. The higher DH proxy values and the SDS-PAGE results support proteolysis during fermentation, which may have contributed to the increased protein extraction efficiency by enhancing the release and solubilization of proteinaceous components from *C. pyrenoidosa*. *B. amyloliquefaciens* fermentation has been reported to improve soluble protein, peptide release, and amino acid-related nutritional properties in protein-rich substrates, such as soybean-based materials and rice beans^25–28^. These findings suggest that FCP may serve as a promising alternative protein source. However, improved digestibility and related nutritional benefits were not directly evaluated in the present study and should be confirmed in future digestion and in vivo studies.

The increased crude protein content in FCP may be attributed to the combined action of cell wall-degrading and proteolytic enzymes produced during fermentation, which likely promoted the disruption of *C. pyrenoidosa* cells and the release of intracellular and proteinaceous components. In addition, the higher crude protein content may also reflect compositional changes in the recovered fraction during fermentation and processing. Accordingly, the crude protein content of FCP and the protein extraction efficiency value should be interpreted as related but distinct measures. Similarly, the observed changes in amino acid composition should be interpreted with caution, because the recovered FCP fraction may contain fermentation-derived soluble components, including *Bacillus*-derived materials. Thus, the amino acid profile reflects the composition of the recovered fermented fraction rather than that of purified algal protein alone. EAAs cannot be synthesized in the body, and an increase in EAA content may improve the nutritional value of *C. pyrenoidosa*. The EAA/TAA ratio of FCP was 42.1%, which is higher than the 40% EAA/TAA ratio reported by the WHO^40^, suggesting that FCP is close to being a complete protein. Branched-chain amino acid (BCAA; valine, isoleucine, leucine) contents significantly increased after fermentation. BCAAs play crucial roles in muscle growth and recovery, suggesting the nutritional potential of FCP^41^. Among the free amino acids, glutamic acid and aspartic acid contribute to an umami taste and enhance the overall flavor of food^42^. Moreover, the detection of γ-aminobutyric acid in FCP suggests possible functional potential arising from fermentation-driven metabolism; however, its physiological relevance and bioactivity should be clarified in future studies.

This interpretation was further supported by Bio-TEM observations. Whereas unFCP retained intact cell walls and dense intracellular structures, FCP showed disrupted cell walls and cellular debris, indicating that fermentation was associated with substantial structural alteration of the *C. pyrenoidosa* biomass^12,38^. Notably, the cellulase-treated sample did not show a distinct structural difference compared with unFCP under the tested conditions, suggesting that cell wall weakening in the present system was not reproduced by the single cellulase treatment alone. Therefore, the improved extraction observed in FCP is more likely to reflect the combined effects of fermentation-associated factors, including microbial growth, extracellular enzyme production, and associated biochemical changes, rather than the action of a single enzyme alone^43^.

Enhancement of antioxidant capacity can be attributed to the conversion of high-molecular-weight proteins into low-molecular-weight peptides and free amino acids by proteolytic enzymes during fermentation. Low-molecular-weight peptides contain more –NH_2_ and –SH groups than high-molecular-weight proteins, allowing them to effectively scavenge radicals^44^. TPC also increased after fermentation, which may be associated with the release of phenolic compounds during cell disruption. However, because the Folin-Ciocalteu assay may also respond to non-phenolic reducing substances and pigments present in the fermented fraction, this increase should be interpreted with caution. The high contents of hydrophobic and aromatic amino acids in FCP also directly affect its antioxidant activity^45^. Wu et al.^28^ reported that the DPPH and ABTS scavenging activities in rice beans (*Vigna umbellata*) increased by 1.9–4.8 times after fermentation with *B. amyloliquefaciens*. Similarly, TPC was increased in soybean meal fermented by *B. amyloliquefaciens* compared to raw or enzymatically hydrolyzed soybean meal^46^. Moreover, *C. pyrenoidosa* protein extracted using microbial protease exhibited high DPPH and ABTS radical scavenging activities^23^. Similar fermentation-associated antioxidant enhancement has also been reported in other microalgal systems, including significantly increased antioxidant activity and total phenolic content in *Arthrospira platensis* after lactic acid fermentation and improved polyphenol content and antioxidant activity in fermented *Chlorella vulgaris* biomass^47,48^. The present results align with these findings. In conclusion, FCP contains abundant polyphenols and has strong antioxidant activity, suggesting its potential as a sustainable protein source.

The characteristic green color of *C. pyrenoidosa* is known to suppress appetite and is generally perceived as unpleasant by consumers^49^. Beyond nutritional and antioxidant advantages, improving sensory acceptability is critical for practical food applications of *C. pyrenoidosa* protein. Because of its unique odor and flavor, *C. pyrenoidosa* can negatively affect consumer perception of food products containing it. Fermentation with *B. amyloliquefaciens* offers a promising approach to address this issue, as the bacterium produces enzymes that break down substances, such as proteins, to generate flavor compounds, creating diverse tastes and aromas^50^. unFCP mainly contained aldehydes and furans, indicating a relatively simple and strong aroma. In contrast, FCP showed a notable increase in alcohols and ketones, leading to a softer and more complex aroma. This change is attributed to the active breakdown of sugars and fatty acid metabolism during fermentation. Aldehydes contribute to the off-odor of microalgae. Notably, hexanal and pentanal, representative compounds associated with the odor of *C. pyrenoidosa*, were not detected in FCP^51^. However, some odor compounds associated with fermentation, such as sulfur dioxide, ethyl n-butyl disulfide, isobutyric acid, and butanoic acid, were also produced. Kurt et al.^52^ reported that the VOC composition in *Spirulina* undergoes significant alterations during fermentation, eliminating off-odor and introducing fermented aromas. In the present study, the VOC composition changed significantly during fermentation, resulting in a complex and rich aroma profile.

In the food industry, various amino acids are used to enhance flavor. Hydrophobic amino acids and BCAAs are associated with bitterness, whereas glutamic acid and aspartic acid contribute to umami flavor^45^. The sensory evaluation results were qualitatively consistent with the observed increases in hydrophobic amino acids, BCAAs, glutamic acid, and aspartic acid after fermentation. Aroma characteristics, such as seaweed, muddy/earthy, and green/grass, are typically considered unsuitable for food products because of their off-flavor^46^. Notably, these undesirable attributes were reduced in FCP. Conversely, the intensity of the “fermented” odor increased, consistent with the VOC results. A limitation of the present VOC analysis is that the results should be interpreted as qualitative changes in volatile profiles rather than quantitative differences in individual compounds. In addition, because the sensory evaluation was conducted using a small student panel and complete blinding was difficult due to visible differences between the samples, further studies using larger and more diverse panels will be needed to strengthen sensory validation.

Finally, the higher values observed at the semi-pilot scale may be due to the direct air injection, which improved oxygen transfer efficiency. Additionally, the precise control over stirring and air injection rates helps maintain a stable culture environment in the fermenter^53^. *B. amyloliquefaciens* is a facultative aerobic bacterium; hence, when supplied effectively, oxygen promotes enzyme production and growth^54^. In *Spirulina* cultivation in a 3 L fermenter, fermentation parameters, such as dissolved oxygen levels, influenced the quality of the final product^55^. In addition, scaling up solid-state fermentation of chicken feathers with *B. subtilis* improved protein extraction efficiency^56^. Salamony et al.^57^ observed increases in the production of soluble protein and alkaline protease during scale-up, which aligns with the findings of the present study^40^. In the present study, the semi-pilot-scale fermentation was conducted under the same validated optimum conditions as the lab-scale fermentation, and similar trends were observed between the two scales. However, because the semi-pilot system included direct air injection and was not operated with direct monitoring of additional process-control parameters, the present results should be interpreted as preliminary evidence of process applicability at a larger scale. Further studies using fermenter-based large-scale production systems will be needed to establish a more controlled and scalable manufacturing process.

This study establishes a scalable bioprocess to produce high-value, water-soluble protein from *C. pyrenoidosa* by combining microbial fermentation (*B. amyloliquefaciens* MU2) with systematic optimization (RSM-BBD). Beyond higher protein extraction efficiencies, the process overcomes the *Chlorella*’s rigid cell wall, generating a fraction rich in low-molecular-weight peptides with improved flavor, enhanced antioxidant activity, and a stronger essential amino acid profile. As a result, the protein is easier to digest and absorb, removing a key barrier to the adoption of *Chlorella* protein. Overall, microbial fermentation substantially improved the qualities of *C*. *pyrenoidosa* protein, positioning it as a promising candidate for sustainable protein applications, although techno-economic feasibility remains to be validated. The approach may be applicable to other biomass-based alternative protein materials, including microalgae and seaweeds and supports a pathway toward a more sustainable protein supply. Future research should focus on systematically optimizing fermentation conditions in larger volumes and applying these methods to industrial-scale production.

## Methods

### Microorganisms and chemicals

*B. amyloliquefaciens* MU2 (KCTC 15499BP; Korea Collection for Type Cultures) was isolated previously by our group from *Jeotgal* collected from Jeju Dongmun Market in South Korea and maintained in glycerol stocks at −80 °C^29^. *B. amyloliquefaciens* MU2 was cultured in All Culture Agar (Sigma-Aldrich, St. Louis, MO, USA) at 37 °C for 24 h. Dried *C. pyrenoidosa* was purchased from Jeongwoodang (Seoul, Korea). All Culture Broth, sodium dodecyl sulfate, acrylamide, methanol, 1,2-bis(dimethylamino)ethane, ABTS, potassium persulfate, DPPH, gallic acid, Folin & Ciocalteu’s phenol reagent, sodium carbonate, and diethyl ether were purchased from Sigma-Aldrich. InstantBlue Coomassie protein stain was purchased from Abcam (Cambridge, MA, USA). HiQ BluePlus Protein Marker was purchased from BioD (Gwangmyeong, Korea). Trichloroacetic acid (TCA) was purchased from Samchun Chemical (Pyeongtaek, Korea). Unless otherwise stated, all materials conformed to analytical grade specifications.

### Fermentation of *C. pyrenoidosa*

*C. pyrenoidosa* was fermented with *B. amyloliquefaciens* MU2 as previously described^58^. In brief, 10 g of dried *C. pyrenoidosa* was resuspended in 200 mL of distilled water by mixing to obtain a 5% (*w/v*) suspension. Carbon (glucose, fructose, and sucrose) and nitrogen (yeast extract, beef extract, and peptone) sources were autoclaved at 121 °C for 15 min and then introduced separately to the *C. pyrenoidosa* medium at a concentration of 1%. *B. amyloliquefaciens* MU2 was cultured on All Culture Broth until 7 log CFU/mL and inoculated at 1% (*v/v*) into the *C. pyrenoidosa* medium. Fermentation was conducted on an orbital shaker (Jeio Tech, Daejeon, Korea) at 37 °C under shaking at 150 rpm for 24 h. The culture was centrifuged at 10,000 × *g* for 20 min (Hanil Scientific, Gimpo, Korea), and the supernatant was collected.

### Analysis of the effects of the fermentation medium composition of *C. pyrenoidosa*

The effects of carbon and nitrogen sources on *C. pyrenoidosa* protein extraction were investigated during and after fermentation by measuring the viable cell count of *B. amyloliquefaciens* MU2 cultured in each nutrient source and the protein extraction efficiency from *C. pyrenoidosa*. The protease activity, cellulase activity and growth of *B. amyloliquefaciens* MU2 were evaluated in media containing only the respective nutrients, without *Chlorella*, to determine the contribution of each component.

To determine the viable cell count, the fermented product was diluted in phosphate-buffered saline and homogenized. One milliliter of the diluted solution was inoculated into All Culture Agar using the pour plate method and incubated at 37 °C for 48 h. A control group was established by inoculating *B. amyloliquefaciens* MU2 into *C. pyrenoidosa* medium without additional nutrients under the same conditions.

Protein extraction efficiency was determined by measuring protein concentrations using the bicinchoninic acid assay (Pierce BCA Protein Assay Kit; Thermo Fisher Scientific, Rockford, IL, USA) as previously described^59^. In a 96-well plate, 200 µL of assay reagent was mixed with 25 µL of sample and the plate was incubated at 37 °C for 30 min. Absorbance was measured at 562 nm using a microplate reader (Synergy HTX Multi-Mode Microplate Reader; BioTek Instruments, Canada). Bovine serum albumin served as the calibration standard for constructing the protein standard curve. Prior to BCA analysis, samples were tested at multiple dilution levels (undiluted, 10×, 20×, 40×, 50×, and 100×). The dilution range used for calculation was selected based on the linear region of the calibration response, and only values within the linear range were used for quantification. To minimize interference from medium-derived proteinaceous components, the BCA responses of yeast extract, beef extract, and peptone were measured separately and subtracted from the corresponding values used for protein extraction efficiency calculation. The protein extraction efficiency was calculated according to Eq. (1):

1 $$\mathrm{Protein}\,\mathrm{extraction}\,\mathrm{efficiency}\,( \% )\,=\,(\mathrm{Pfcp})/\mathrm{Pcp}\,\times \,100$$

where Pcp is the initial protein concentration of the *C. pyrenoidosa* slurry on the same broth-volume basis (mg/mL) and Pfcp is the protein concentration measured in the recovered FCP fraction.

Protease activity was measured using casein as the substrate, following a previously described method^60^. The cell-free culture supernatant of *B. amyloliquefaciens* MU2 grown under each nutrient condition was used as the crude extracellular enzyme source. A 0.5 mL aliquot of 1% (*w/v*) casein solution and 0.5 mL enzyme solution were each prewarmed at 30 °C and then mixed. The mixture was incubated at 30 °C for 10 min, after which 1 mL of 400 mM TCA was added to terminate the reaction. Following centrifugation at 13,000 × *g* for 5 min, the supernatant was collected. A 1 mL aliquot of the supernatant was combined with 5 mL of 400 mM Na_2_CO_3_ and 1 mL of Folin & Ciocalteu’s phenol reagent, and the mixture was incubated at 30 °C for 20 min. Absorbance was then measured at 680 nm using the microplate reader. One unit was defined as the enzyme amount hydrolyzing casein to produce 1 μg of tyrosine per minute.

Cellulase activity was evaluated as carboxymethyl cellulase activity using the dinitrosalicylic acid method with slight modification of a previously reported procedure^61^. To compare the cellulolytic potential of *Bacillus amyloliquefaciens* MU2 under different nutrient-source conditions, MU2 was cultured in medium containing 1% (w/v) carboxymethyl cellulose together with the indicated nutrient source under the same temperature and incubation period used for the screening experiment. After incubation at 37 °C for 24 h, the culture broth was centrifuged, and the supernatant was collected as the extracellular enzyme source. The reaction mixture consisted of 250 μL of enzyme supernatant and 250 μL of 1% (w/v) CMC dissolved in 0.05 M sodium phosphate buffer (pH 7.0), followed by incubation at 37 °C for 15 min. The reaction was terminated by adding 500 μL of DNS reagent and heating the mixture in boiling water. Absorbance was measured at 540 nm, and cellulase activity was calculated from a glucose standard curve. One unit (U) of CMCase activity was defined as the amount of enzyme required to release 1 μmol of reducing sugar per min under the assay conditions.

The growth kinetics of *B. amyloliquefaciens* MU2 were determined during culture in media supplemented with yeast extract, beef extract, or peptone at 37 °C under rotation at 150 rpm for 30 h. Absorbance (600 nm) was measured at 30-min intervals using a microplate reader.

### Experimental design and optimization for fermentation conditions

The Design-Expert Software version 13.0 (Stat-Ease, Minnesota, MN, USA) was used to establish optimal fermentation conditions for protein extraction from *C. pyrenoidosa*. RSM-BBD was employed to create models with fermentation time (*A*, h), fermentation temperature (*B*, °C), and inoculation rate (*C*, %) as the independent variables. The protein extraction efficiency (%) from *C. pyrenoidosa* was selected as the dependent variable. Fermentation time, temperature, and inoculation rate were selected as independent variables because preliminary single-factor experiments indicated that these factors had marked effects on protein extraction efficiency and were practically controllable during fermentation. The tested ranges were determined based on the preliminary results (Fig. S1), in which stable growth of *B. amyloliquefaciens* MU2 and measurable differences in protein extraction efficiency were observed. The ranges selected for optimizing the fermentation conditions for *B. amyloliquefaciens* MU2 were as follows: fermentation time (A), 18–30 h, fermentation temperature (B), 25–45 °C, and inoculum concentration (C), 1–5%. The design comprised 17 runs with five central point replications to estimate pure error and assess repeatability. A quadratic response surface was fitted by least squares and evaluated by ANOVA, followed by numerical optimization using a desirability function. Model predictions were verified by triplicate confirmation runs at the computed optimum, comparing the observed protein extraction efficiency with the model prediction. For subsequent analyses, unFCP refers to the unfermented *C. pyrenoidosa* biomass, whereas FCP refers to the centrifugation-recovered supernatant fraction of fermented *C. pyrenoidosa* obtained under the optimized conditions and freeze-dried for use in experiments (Fig. 3).

### Proximate composition of unFCP and FCP

Moisture content was determined by measuring the weight before and after air drying at 105 °C. Ash content was determined after dry ashing at 550 °C. Crude protein content was determined using a Kjeldahl analyzer (Kjeltec 8400; FOSS, Hoganas, Sweden). It was calculated from total nitrogen using a conversion factor of 6.25. Crude fat content was determined using Soxhlet extraction (Soxtec 2055 Fat Extraction System; FOSS). Diethyl ether was used as the extraction solvent, and the solvent was removed on a water bath at 50–60 °C before gravimetric determination.

### Amino acid composition of unFCP and FCP

The total and free amino acid contents of unFCP and FCP were determined as previously described^62^. Samples were hydrolyzed with 10 mL of 6 N HCl at 110°C for 24 h. Then, HCl was concentrated using an evaporator (Hei VAP Expert, Heidolph, Schwabach, Germany) and diluted with 0.02 N HCl solution for total amino acid analysis. For free amino acid analysis, the samples were combined 1:1 with 16% TCA, shaken for 15 min. The mixture was centrifuged (3000 × *g*, 15 min), and the supernatant was collected. The total and free amino acids were quantified by an automatic analyzer (L-8900; Hitachi High-Technologies, Tokyo, Japan) equipped with a Hitachi ion-exchange HPLC packed column (4.6 mm i.d. × 60 mm, 3 μm). Total and free amino acid contents are expressed as mg/100 g dry weight of the analyzed fraction.

### Degree of hydrolysis analysis of unFCP and FCP

The DH of unFCP and FCP was determined using the trichloroacetic acid (TCA) precipitation method^63^. One milliliter of 20% TCA was added to 1 mL of the sample and incubated at 4 °C for 30 min. The mixture was centrifuged (10,000 × *g*, 15 min) to remove impurities and insoluble proteins. DH was calculated using the following Eq. (2):

2 $$\mathrm{DH} \% =\mathrm{Ps}/\mathrm{Pt}\times 100$$

where Ps is the TCA-soluble protein content of the fraction being analyzed and Pt is the total protein content of that fraction. Thus, DH of unFCP was calculated from the TCA-soluble and total protein contents of unFCP, whereas DH of FCP was calculated from the TCA-soluble and total protein contents of FCP.

### Bio-TEM

Cell wall degradation in *C. pyrenoidosa* was examined by bio-transmission electron microscopy (Bio-TEM) using a Talos L120C TEM (Thermo Scientific, USA). Three sample groups were prepared for microscopic observation: unfermented *C. pyrenoidosa* biomass (unFCP), fermented *C. pyrenoidosa* fraction (FCP), and a cellulase-treated sample. The cellulase-treated sample was prepared by suspending dried *C. pyrenoidosa* biomass in distilled water to obtain a 5% (w/v) slurry, followed by treatment with cellulase from Trichoderma reesei (700 U/g; Sigma-Aldrich, USA) at 3% (w/w, substrate basis) at pH 5.0 and 50 °C for 2 h^23^. For Bio-TEM imaging, wet samples were fixed in a 2% paraformaldehyde–glutaraldehyde mixture buffered with 0.05 M sodium cacodylate (pH 7.2) for 2 h. Following fixation, samples were treated for 1 h with 1% osmium tetroxide in the same buffer. The samples were then dehydrated through a graded ethanol series and propylene oxide, followed by infiltration with Embed 812 resin. The prepared sections were stained with 2.5% uranyl acetate and lead citrate. Cross-sectional images of the Chlorella samples were observed at an accelerating voltage of 120 kV.

### SDS-PAGE analysis of unFCP and FCP

Protein fractions were determined using SDS-PAGE with a Mini-Protein Bio-Rad electrophoresis system (Bio-Rad Laboratories, Watford, UK). Electrophoresis was performed using a 12% separating gel and a 5% stacking gel^64^. The samples were combined with 5× SDS-PAGE sample buffer at a 4:1 (*v/v*) ratio and heated at 100 °C for 10 min. Twenty microliters of each sample was loaded into the wells and electrophoresed at 110 V for 1.5 h. The gel was stained and destained using InstantBlue Coomassie Protein Stain (ab119211; Dawin Bio Inc., Korea). The fractions were identified using the HiQ BluePlus Protein Marker (A20028, BioD), with a mass range of 10–245 kDa.

### Assessment of antioxidant activity and total phenolic content

ABTS radical scavenging activity was determined following previously described^65^, with minor adjustments. An ABTS stock (7 mM) was generated with 2.4 mM potassium persulfate and then diluted to Abs_734_ of 0.700 ± 0.005. Then, equal volumes (500 μL) of sample and the ABTS solution were reacted for 6 min in the dark at room temperature, and absorbance was measured at 734 nm using a microplate reader. Scavenging activity was calculated according to the following Eq. (3):

3 $$\,\mathrm{ABTS}\,\mathrm{scavenging}\,\mathrm{activity}\,( \% )\,=\,({\mathrm{Abs}}_{\mathrm{control}}\,-\,{\mathrm{Abs}}_{\mathrm{sample}})/{\mathrm{Abs}}_{\mathrm{control}}\,\times \,100$$

DPPH radical scavenging activity was evaluated as previously reported^66^. Briefly, 1 mL of 0.15 mM DPPH solution was combined with 0.5 mL of the sample, and the mixture was shaken and kept in the dark at room temperature for 30 min, and absorbance was measured at 517 nm using a microplate reader. Scavenging activity was calculated as the following Eq. (4):

4 $$\,\mathrm{DPPH}\,\mathrm{scavenging}\,\mathrm{activity}\,( \% )\,=\,({\mathrm{Abs}}_{\mathrm{control}}\,-\,{\mathrm{Abs}}_{\mathrm{sample}})/{\mathrm{Abs}}_{\mathrm{control}}\,\times \,100$$

TPC was determined as previously described^67^, with some modifications. 1 mL of 1 N Folin & Ciocalteu’s phenol reagent was added to 200 μL of the sample and allowed to react for 3 min at 20 ± 5 °C. Subsequently, 2 mL of 10% (*w/v*) Na_2_CO_3_ was added and reacted for 30 min in the dark at room temperature. After centrifugation (15,928 × *g*, 5 min), 200 μL of the supernatant was transferred to a 96-well plate, and absorbance was measured at 760 nm using a microplate reader. Gallic acid standard prepared by 2-fold serial dilution was used to construct the calibration curve, and the results were expressed as gallic acid equivalent (μM GAE/mL).

### Analysis of the sensory properties of unFCP and FCP

The sensory evaluation was conducted by 20 trained students majoring in Food Science and Technology at Pukyung National University. Each panelist assessed unFCP and FCP in triplicate for basic taste attributes (sweet, salty, astringency, bitter, and umami), and flavor attributes (seaweed, fermented, green/grass, muddy/earthy). The green/grass and muddy/earthy descriptors were selected because these notes are commonly recognized as undesirable sensory characteristics of microalgal biomass, including *Chlorella* spp^68^. The panelists quantified the features using a 5-point intensity scale (1 = very weak, 5 = very strong). Samples were presented in randomized order, although complete blinding was not feasible because of visible differences. All participants provided written informed consent prior to testing; the protocol was approved by the Institutional Review Board of Pukyung National University (1041386-202407-HR-90-01) and adhered to the Declaration of Helsinki.

VOCs in unFCP and FCP were identified using headspace SPME coupled with GC-MS (Agilent 7890B-7000C; Agilent Technologies, USA) as described previously^69^. For VOC analysis, the analyzed powder-form sample was placed in the headspace vial and equilibrated under the conditions described below. The samples were equilibrated at 60 °C for 15 min with agitation, after which a SPME fiber (Supelco 57306, USA) was exposed to the vial headspace at 60 °C for 20 min. The fiber was then introduced into the GC inlet and thermally desorbed at 210 °C for 10 min. Analyses were conducted in splitless mode. The oven programme started at 35 °C (10 min hold) and was ramped to 200 °C at 5 °C/min. Helium served as the carrier gas at 1.0 mL/min. Temperatures for the injection port, ion source, and quadrupole were 240 °C, 230 °C, and 150 °C, respectively. Mass spectra were acquired in EI mode at 70 eV, scanning 20–350 m/z. Volatile compounds were tentatively identified using the NIST mass spectral library. Only compounds with a library match similarity of over 80% were selected, and odor descriptors were assigned according to the Flavornet database (https://www.flavornet.-org/f_kovats.html).

### Semi-pilot-scale experiment

A semi-pilot-scale experiment was conducted using a 7-L bioreactor (FMT ST-D; Fermentech, Korea) with a working volume of 3 L. To prepare the medium, 150 g of dried *C. pyrenoidosa* was mixed with 3 L of 1% yeast extract media, resulting in a 5% medium. *B. amyloliquefaciens* MU2 was cultured in All Culture Broth to approximately 7 log CFU/mL and inoculated into the *C. pyrenoidosa* medium at the same inoculation rate used under the optimized conditions (2.7%, v/v). Fermentation was carried out under the validated optimum conditions (33.8 °C, 26.6 h) with stirring at 250 rpm and air injection (1 L/min). Lab-scale fermentation was conducted under the same optimized conditions in a 200 mL Erlenmeyer flask, except for direct air injection. Following fermentation, the supernatant of culture medium was collected by centrifuging (10,000 × *g* for 20 min) for protein extraction efficiency analysis as described above. Additionally, the recovered supernatant was lyophilized to assess weight recovery rate, which was calculated using the following Eq. (5):

5 $$\mathrm{Weight}\,\mathrm{recovery}\,\mathrm{rate}\,( \% )\,=\,\mathrm{weight}\,\mathrm{of}\,\mathrm{recovered}\,\mathrm{supernatant}\,\mathrm{fermented}\,C.\,pyrenoidosa\,(g)/\mathrm{weight}\,\mathrm{of}\,\mathrm{initial}\,C.\,pyrenoidosa\,(g)$$

### Statistical analysis

Data are presented as mean ± SD in triplicate. Statistical analyses were conducted in SPSS 27 (IBM SPSS Advanced Statistics, Chicago, IL, USA). For comparisons among more than two groups, one-way ANOVA followed by Duncan’s multiple range test was used. For two group comparisons, an independent samples t-test was used. Statistical significance was set at *p* < 0.05

## Supplementary information

Supplementary Information

Supplementary file_raw data set

## Data Availability

The data supporting the findings of this study are included in this article and its Supplementary Information files.
