# Supplementary material for: Bacillus amyloliquefaciens MU2 fermentation enhances protein extraction and sensory quality of Chlorella pyrenoidosa
Source: NPJ Sci Food. 2026 Jun 20;10:306. doi: 10.1038/s41538-026-00949-7 (PMC13645729; doi:10.1038/s41538-026-00949-7)
Supplement: Supplementary file 1 [file 41538_2026_949_MOESM1_ESM.pdf]

Supplementary information

Supplementary Figure S1. Protein extraction efficiencies from *Chlorella pyrenoidosa* fermented with *Bacillus amyloliquefaciens* MU2 in function of (A) fermentation time (h), (B) fermentation temperature (°C), and (C) inoculation rate (%). Values with different letters vary significantly ($p < 0.05$).

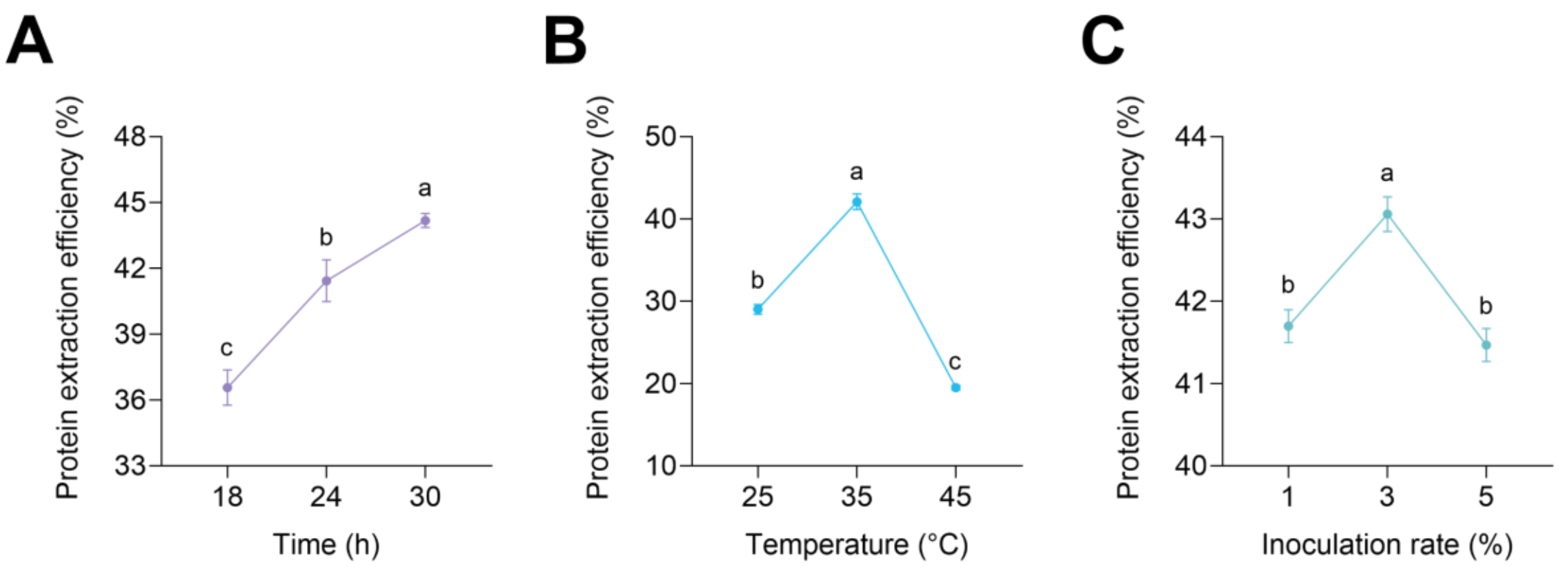

Supplementary Figure S2. Residual diagnostic plots for model adequacy. (A) Normal plot of residuals; (B) residuals versus predicted; (C) residuals versus run order.

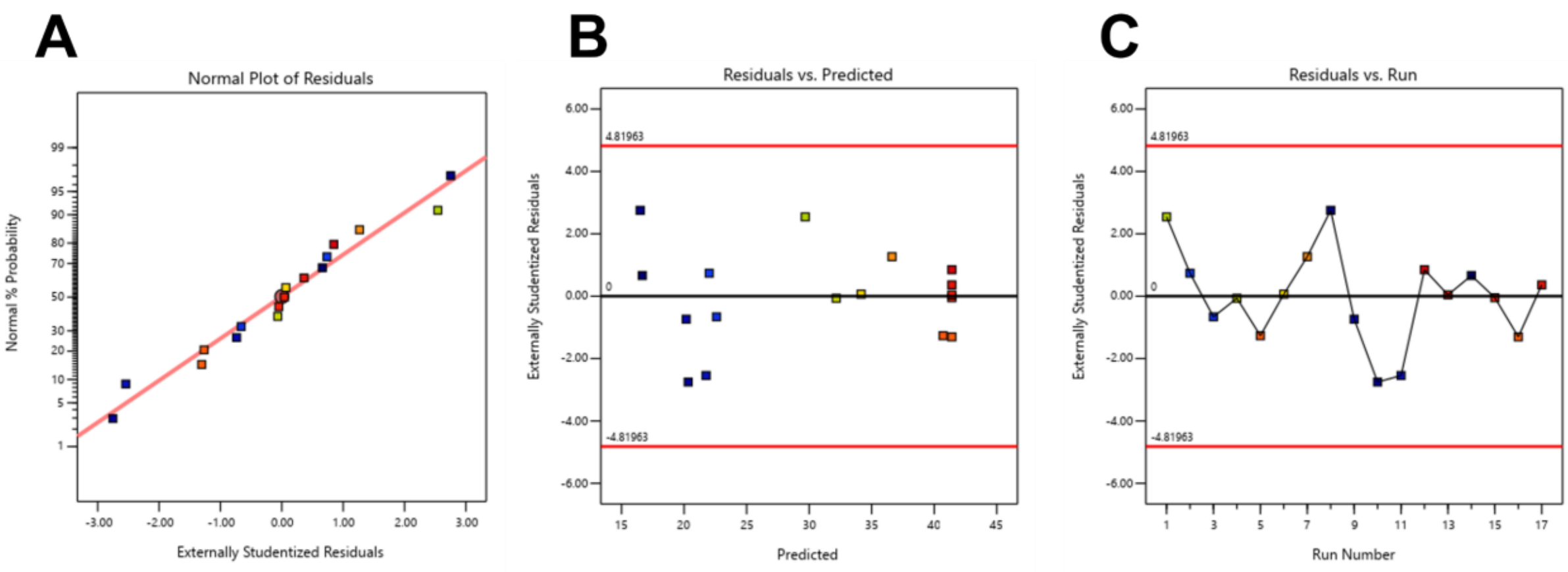

Supplementary Table S1. Box–Behnken experimental design and protein extraction efficiency from *C. pyrenoidosa*

No.	A, Fermentation time (h)	B, Fermentation temperature (℃)	C, Inoculation rate (%)	Y, Protein extraction efficiency (%)
1	30	25	3	31.36
2	18	25	3	22.68
3	24	25	1	21.99
4	18	35	1	32.10
5	30	35	1	39.65
6	30	35	5	34.22
7	18	35	5	37.69
8	24	45	1	18.23
9	30	45	3	19.50
10	24	25	5	18.60
11	18	45	3	20.09
12	24	35	3	42.76
13	24	35	3	41.48
14	24	45	5	17.27
15	24	35	3	41.32
16	24	35	3	39.46
17	24	35	3	42.01

Supplementary Table S2. Analysis of variance and adequacy of the quadratic model

**Source**	**Sum of squares**	**Degree of freedom**	**Mean square**	***F*-value**	***P*-value**
Model	1304.96	9	145.00	24.78	< 0.0001 Significant
A	5.34	1	5.34	0.91	0.0440
B	74.22	1	74.22	12.68	0.0056
C	0.17	1	0.17	0.03	0.0426
AB	21.44	1	21.44	3.66	0.0334
AC	50.89	1	50.89	8.70	0.0163
BC	13.61	1	13.61	2.33	0.5108
A^2	15.07	1	15.07	2.58	0.5390
B^2	1011.81	1	1011.81	172.90	< 0.0001
C^2	61.04	1	61.04	10.43	0.0007
Residual	40.96	7	5.85		
Lack of Fit	32.43	3	10.81	5.06	0.1327 Not significant

Supplementary Table S3. Regression analysis for the quadratic model.

Source	Predicted value (%)
Mean	30.61
Standard deviation	1.75
Model degree	Quadratic
R^2 (Adj R^2)	0.9864 (0.9689)
Adequacy precision	18.53

Supplementary Table S4. Validation of the optimized model of fermentation conditions for *Chlorella* protein extraction by *B. amyloliquefaciens* MU2.

**Dependent variable**	**Actual experiment variables**	**Predicted value (%)**	**Actual value (%)**	**Error (%)**
Protein extraction efficiency	A, fermentation time 26.6 h<br>B, fermentation temperature 33.8℃<br>C, inoculation rate 2.7%	42.98	42.9	0.18

Supplementary Table S5. Proximate composition of unFCP and FCP

Proximate composition	unFCP	FCP
Carbohydrate	28.60 ± 0.26	6.03 ± 0.21
Crude protein	58.47 ± 0.21	78.10 ± 0.20
Crude fat	4.03 ± 0.06	1.67 ± 0.06
Moisture	3.33 ± 0.15	7.00 ± 0.10
Ash	5.57 ± 0.06	7.20 ± 0.00

Supplementary Table S6. Total amino acid composition of unFCP and FCP.

Amino acids	Concentration of total amino acid (mg/100 g dry basis)		*t*-Value	*p*-Value
	unFCP	FCP		
Aspartic acid	3847.4 ± 26.4	4892.9 ± 32.9	-31.308	0.001
Threonine*	2036.0 ± 32.9	2552.2 ± 35.1	-14.331	0.005
Serine	1748.6 ± 35.1	1868.1 ± 54.2	-2.433	0.135
Glutamic acid	5032.5 ± 26.8	6539.8 ± 35.6	-64.797	< 0.001
Glycine	2539.1 ± 58.9	3234.9 ± 61.4	-30.113	0.001
Alanine	3763.1 ± 36	3903.4 ± 41.2	-8.917	0.012
Cystine	533.4 ± 13.7	625.0 ± 14.5	-155.270	< 0.001
Valine*	2813.2 ± 40.6	3538.3 ± 26.5	-55.538	< 0.001
Methionine*	945.9 ± 11.1	1208.5 ± 18.3	-36.855	0.001
Isoleucine*	1770.8 ± 44.3	2320.9 ± 33.3	-51.543	< 0.001
Leucine*	4161.3 ± 61.1	4919.0 ± 42.1	-69.069	< 0.001
Tyrosine	1586.0 ± 16.2	1798.7 ± 21.2	-9.862	0.010
Phenylalanine*	2473.6 ± 42	2677.1 ± 47.1	-7.144	0.019
Lysine*	3717.1 ± 22.5	4016.5 ± 50.0	-7.223	0.019
Histidine	923.4 ± 10.5	1190.4 ± 12.1	-254.190	< 0.001
Arginine	2801.0 ± 47.4	3159.7 ± 41.3	-7.015	0.020
Proline	2342.4 ± 43.4	3401.1 ± 43.5	-21.296	0.002
∑EAA	17917.9 ± 118.9	21232.5 ± 252.4	-42.176	0.001
∑NEAA	25116.9 ± 73.0	30614.0 ± 357.9	-22.924	0.002
TAA	43034.8 ± 69.8	51846.5 ± 610.3	-27.839	0.001

Supplementary Table S7. Free amino acid composition of unFCP and FCP.

Amino acids	Concentration of total amino acid (mg/100 g dry basis)		*t*-Value	*p*-Value
	unFCP	FCP		
Phosphoserine	38.2±0.2	418.8±3.5	-190.221	< 0.001
Urea	248.1±0.8	357.6±3.8	-44.179	< 0.001
Aspartic acid	13.5±0.1	232.0±1.8	-222.142	< 0.001
Threonine*	48.1±0.6	332.3±1.3	-260.435	< 0.001
Serine	17.4±0.2	11.3±0.0	45.5	< 0.001
Glutamic acid	284.3±3.5	548.1±10.7	-55.934	< 0.001
Sarcosine	0.0±0.0	151.4±9.6	-27.449	0.001
α-amino adipic acid	0.0±0.0	20.3±0.6	-56.302	< 0.001
Proline	119.3±0.6	555.5±5.4	-150.743	< 0.001
Glycine	24.0±1.1	154.6±4.9	-58.897	< 0.001
Alanine	163.0±2.8	207.5±20.0	-4.455	0.047
Citrulline	0.0±0.0	28.9±1.0	-28.9	0.022
Valine*	29.7±0.5	950.6±25.9	-62.1	< 0.001
Cystine	2.3±0.1	130.6±0.8	-277.201	< 0.001
Methionine*	7.9±0.1	270.8±10.1	-45.261	< 0.001
Cystathionine	0.0±0.0	54.6±3.7	-25.789	0.002
Isoleucine*	11.6±0.1	676.5±14.7	-77.913	< 0.001
Leucine*	16.7±0.2	1447.8±107.0	-23.176	0.002
Tyrosine	6.8±0.1	267.5±20.0	-22.441	0.002
Phenylalanine*	23.4±0.1	980.8±16.6	-100.313	< 0.001
β-alanine	3.3±0.1	287.2±15.4	-31.876	< 0.001
β-Amino isobutyric acid	0.0±0.0	391.7±7.6	-88.982	< 0.001
γ-Amino-n-butyric acid	10.2±0.5	115.7±16.0	-11.131	0.009
Hydroxylysine	10.4±0.3	26.7±2.7	-10.514	0.008
Ornithine	7.3±0.2	191.3±11.8	-27.462	0.001
Lysine*	72.8±0.4	704.9±13.5	-83.184	< 0.001
Arginine	34.4±0.7	102.6±3.5	-42.377	< 0.001
∑EAA	210.1±0.3	5363.8±151.7	-58.969	< 0.01
∑NEAA	982.4±8.4	4254.1±65.5	-97.115	< 0.01
TAA	1192.5±8.7	9617.9±215.2	-69.951	< 0.01
